# Moderate Genetic Diversity of MHC Genes in an Isolated Small Population of Black-and-White Snub-Nosed Monkeys (*Rhinopithecus bieti*)

**DOI:** 10.3390/ani14152276

**Published:** 2024-08-05

**Authors:** Jibing Yan, Chunmei Song, Jiaqi Liang, Yanni La, Jiandong Lai, Ruliang Pan, Zhipang Huang, Baoguo Li, Pei Zhang

**Affiliations:** 1 Shaanxi Key Laboratory of Animal Conservation, College of Life Sciences, Northwest University, Xi’an 710069, China; yjibingbing@163.com (J.Y.); m18821657732@163.com (C.S.); liangjiaqi0508@163.com (J.L.); lalayannini@163.com (Y.L.); ruliang.pan@uwa.edu.au (R.P.); 2 Baima Snow Mountain National Nature Reserve Administrative Bureau, Diqing 674500, China; 18987296863@163.com; 3 International Center of Biodiversity and Primat Conservation, Dali University, Dali 671003, China; 4 School of Human Sciences, The University of Western Australia, Perth, WA 6009, Australia; 5 Institute of Eastern-Himalaya Biodiversity Research, Dali University, Dali 671003, China; huangzp@eastern-himalaya.cn; 6 Shaanxi Institute of Zoology, Xi’an 710032, China; 7 College of Life Science, Yanan University, Yanan 710032, China

**Keywords:** *Rhinopithecus bieti*, genetic diversity, MHC, balancing selection

## Abstract

**Simple Summary:**

Genetic diversity plays a crucial role in determining the ability of populations to evolve. We investigated the genetic variation of the black-and-white snub-nosed monkey by integrating adaptive MHC genes and neutral microsatellites. We found that neutral loci exhibited high heterozygosity and a high degree of polymorphism, while MHC genes showed high heterozygosity and moderate polymorphism. Additionally, positive selection and trans-species evolution indicated that historical balancing selection might have sustained the MHC polymorphism. This study provides valuable scientific evidence and a reference for formulating or amending conservation strategies for black-and-white snub-nosed monkeys.

**Abstract:**

Genetic diversity is an essential indicator that echoes the natural selection and environmental adaptation of a species. Isolated small populations are vulnerable to genetic drift, inbreeding, and limited gene flow; thus, assessing their genetic diversity is critical in conservation. In this study, we studied the genetic diversity of black-and-white snub-nosed monkeys (*Rhinopithecus bieti*) using neutral microsatellites and five adaptive major histocompatibility complex (MHC) genes. Two *DQA1* alleles, two *DQB1* alleles, two *DRB1* alleles, two *DRB5* alleles, and three *DPB1* alleles were isolated from a population. The results indicate that neutral microsatellites demonstrate a high degree of heterozygosity and polymorphism, while adaptive MHC genes display a high degree of heterozygosity and moderate polymorphism. The results also show that balancing selection has prominently influenced the MHC diversity of the species during evolution: (1) significant positive selection is identified at several amino acid sites (primarily at and near antigen-binding sites) of the *DRB1*, *DRB5*, and *DQB1* genes; (2) phylogenetic analyses display the patterns of trans-species evolution for all MHC loci. This study provides valuable genetic diversity insights into black-and-white snub-nosed monkeys, which dwell at the highest altitude and have experienced the harshest environmental selection of all primates globally since the Pleistocene. Such results provide valuable scientific evidence and a reference for making or amending conservation strategies for this endangered primate species.

## 1. Introduction

The crucial role of genetic diversity in determining a population’s evolutionary viability has been recognized in recent decades [1]. Loss of genetic diversity in a species can lead to a decline in individual fitness and the constraining of prospective development [2], expressed by decreasing reproductive success and offspring survival [3,4], increasing susceptibility to disease and parasites [5,6], reducing longevity [7], and so on. Factors including inbreeding, genetic drift, restricted gene flow, and small population sizes contribute to reduced genetic diversity [8,9]. Isolated small populations are especially vulnerable to these factors, which can quickly drive the species into an extinction spiral [10]—they suffer from gene drift and inbreeding, which reduce survival fitness directly via increased genetic load and indirectly via erosion of genetic variation, and reduce the adaptability of prospective development, finally driving them to a smaller population until extinction [11]. Thus, dynamic monitoring of genetic diversity for a given species is required to provide its genetic information, used in making conservation plans.

The assessment commonly employed in genetic diversity for wildlife populations is neutral genetic markers, such as the mitochondrial control region (D-loop) and nuclear short tandem repeats (SSRs and microsatellites), whose variations are greatly influenced by population history and genetic drift but less affected by selection pressures [12]. However, population history, genetic drift, and natural selection collectively influence adaptive genetic variation. Thus, neutral genetic markers cannot reveal how a population copes with environmental changes [13]. Therefore, to effectively assess such genetic diversity, it is necessary to combine neutral and adaptive genetic markers.

Among many others, the major histocompatibility complex (MHC) gene family is an ideal candidate for adaptive markers. It is highly polymorphic and unique to vertebrates, playing an essential role in the immune system. Its translated cell surface glycoproteins can recognize and present antigens to T lymphocytes, thus triggering an appropriate immune response [14,15]. According to the different antigens that MHC molecules present, MHC molecules can be divided into two classes: I, on the surface of almost all somatic cells, is responsible for presenting endogenous antigens (e.g., viruses) to cytotoxic CD8^+^ T cells [14,15,16]; II, only in antigen-presenting cells, presents exogenous antigens (e.g., bacteria and parasites) to helper CD4^+^ T cells [14,15,16]. Therefore, the MHC sequences identified in many species, especially their antigen-binding sites (ABSs), determine the range of pathogens an individual can resist, making these loci crucial for disease resistance and subject to pathogen-mediated balancing selection [17,18,19,20,21,22].

The black-and-white snub-nosed monkey (*Rhinopithecus bieti*) is one of the endangered primate species according to the IUCN Red List [23]. It is only distributed in a narrow area between the Lancang and Jinsha Rivers in the Yunling Mountains, featuring fragmented populations. Its populations are in coniferous, mixed coniferous, and broad-leaved forests at an altitude of 3000–4000 m, where no other primates reach [24,25,26,27]. Establishing a reserve for this species has led to a population increase, from less than 2000 to nearly 3000 over the past 25 years [28]. However, fragmentation is the primary threat in some populations following increasing human activities, reducing their size and pushing them to extinction [27,28].

The species features a multilevel society (MLS), compromising several one-male units (OMUs) and at least one all-male unit (AMU) [29,30]. Each OMU consists of one adult male, multiple adult females, subadult individuals and infants, while AMUs include adult, sub-adult, and juvenile males [31].

Previous genetic studies on the species have included using neutral markers (the D-loop and microsatellites) and whole genome sequencing to investigate genetic diversity, evolutionary history, and phylogeography [32,33,34,35,36,37]. *R. bieti* has high genetic diversity, and human activities have severely hindered gene flow between populations, referring to the results from the amplification of 10 microsatellite loci from 135 individuals of eleven populations [35]. A previous study indicate that among the five species of snub-nosed monkeys (*R. bieti*, *R. roxellana*, *R. brelichi*, *R. strykeri*, *and R. avunculus*), *R. bieti* possesses the lowest level of whole genome heterozygosity (*H*_E_ = 0.034%) [33]. Meanwhile, the adaptive genetic diversity of *R. bieti* remains largely unknown.

Thus, in this study, we aimed to (1) investigate the genetic diversity level in *R. bieti*, based on nine neutral microsatellite loci and five adaptive MHC loci (*DQA1*, *DQB1*, *DPB1*, and two *DRB* genes) and (2) identify different selection agents in maintaining MHC diversity of the *R. bieti.*

## 2. Methods

### 2.1. Study Site and Sampling

This study was carried out on the *R. bieti* population inhabiting the Xiangguqing (XGQ) ecotourism area, Baima Snow Mountain Reserve, Yunnan Province (27°36′ N, 99°15′ E), from September to December 2020. During the research period, 57–62 individuals were observed from 8–10 OMUs and one AMU. We collected 52 fecal samples which were stored in 50 mL DET solution (20% DMSO, 0.25 M sodium-EDTA, 100 mM Tris–HCl, pH 7.5, and NaCl to saturation) and kept at −20 °C. They were collected within 15 min of excretion, without being invasive to the monkeys.

### 2.2. DNA Extraction

Genomic DNA was extracted from each fecal sample using QIAamp DNA Stool Mini Kits (Qiagen, Hilden, Germany). Because of the relatively low quality of genetic samples, DNA extraction and subsequent polymerase chain reactions (PCRs) were performed in an ultra-clean laboratory. The facilities were washed with 75% ethanol, and finally UV lamps were used for at least two hours to destroy any residual DNA. All apparatus, including glassware and plastic-ware, was exposed to UV light for 30 min to eliminate any potential contamination by human DNA. Blank controls (without template DNA) were also performed for both extractions and amplifications, and all batches with the negative control amplification products were discarded to prevent unintentional human DNA contamination.

### 2.3. Microsatellite Genotyping

We collected 17 microsatellite loci by pre-experiment through a literature review [38,39], selected 11 that could be successfully amplified, and then synthesized fluorescently labeled primers (Table S1). Among the 11 loci, two loci (*D8S505* and *D2S1326*) were abandoned because the peak profiles of capillary electrophoresis were messy and difficult to identify. To eliminate genotyping errors caused by false alleles and allelic dropouts, all heterozygotes were confirmed for three replicates, and all homozygotes were confirmed for at least seven replicates [40]. After individual identification, 48 non-repetitive individuals were identified from 52 sampled individuals.

### 2.4. MHC Genotyping

Based on the second exon regions of the *DQB* gene (GenBank accession number: NW_016813514.1) and *DRB* gene (GenBank accession number: NW_016819733.1) in *R. bieti*, we designed two pairs of consensus primers to amplify the second exons of *DQB1* (F: 5′-TCCCCGCAGAGGATTTCGTG-3′; R: 5′-AAGGCGACGACACTCACCTC-3′) and *DRB* (F: 5′- GCCCCTGTGACCGGATCGTT-3′; R: 5′-TCCCAGCTCACAGGGACCCAG-3′). To amplify the second exons of *DQA1* and *DPB1*, we used two pairs of primers from *R. roxellana* (F’: 5′-TTYTTTCTTCCCCTGTTCTCC-3′; R’: 5′- TGAAAYTTGGTATGAAGGGATAGA-3′) (modified from [41]) and *Macaca mulatta* (F: 5′-TGAGAGTGGCGCCTCCGCTCAT-3′; R: 5′-AGCCCGGCCCAAAGCCTCACTC-3′) [42], respectively. Genotyping of *DQA1*, *DQB1*, and *DPB1* was conducted by cloning and sequencing 12 clones for each individual. As for *DRB*, we amplified two loci using a pair of primers and conducted amplicon-based next-generation sequencing (NGS).

The PCR was carried out in a 50 μL solution including 10–100 ng of genomic DNA, 0.4 μM of forward and reverse primers (barcode incorporation primers were used for *DRB*), 50 mM KCl, 10 mM Tris-HCl (pH 8.4), 2.5 mM MgCl_2_, 0.2 mM of each dNTP, and 1 unit of ExTaq DNA polymerase (Takara, Dalian, China). Amplification was carried out in a Veriti™ 96-Well Fast Thermal Cycler (Applied Biosystems, Singapore) under the following conditions: initial denaturation at 94 °C for 5 min, followed by 35 cycles of denaturation at 94 °C for 30 s, annealing at appropriate temperature (*DQA1*: 56 °C; *DQB1*: 59 °C; *DPB1*: 68 °C; *DRB*: 58 °C) for 30 s and extension at 72 °C for 30 s, finishing with a final extension at 72 °C for 10 min. Amplified products were purified using an AxyPrep™ DNA Gel Extraction Kit (AXYGEN Biosciences, Union City, CA, USA) according to the manufacturer’s instructions. For *DQA1*, *DQB1*, and *DPB1*, the purified PCR products were then ligated into a pMD 18-T Vector (Takara) and transformed into a DH5α competent cell (Takara) following the manufacturer’s instructions. Twelve positive clones containing inserts from each amplified product were sequenced in both directions with an ABI-PRISMTM 3100 Genetic Analyzer (Applied Biosystems Inc.). Chromas V2.6.6 was used to align all sequences. We defined any sequence as an allele if it was detected in at least two individuals. The purified PCR products for *DRB* were quantified with a Qubit high-sensitivity kit and normalized to meet a final concentration of 10 ng/μL in a mixed amplicon library. Subsequently, the library was sequenced on an Illumina NovaSeq 6000 platform with 250 bp pair-end reads at Beijing Novogene in Beijing, China. The raw fastq files were processed using a bioinformatics pipeline described previously, facilitating the accurate identification of true alleles while excluding artifacts [43,44]. The work-flow was composed of the following four steps: (1) the preparation of raw files for processing; (2) the initial data quality inspection and read filtering; (3) the identification of putative MHC alleles and artifacts; and (4) the assignment of alleles to individuals [43]. MHC-TYPER V1.0 [45] was then used to assign *DRB* alleles to a specific locus.

In addition, we blasted the obtained MHC sequences on the NCBI (https://www.ncbi.nlm.nih.gov, accessed on 28 November 2021). If the obtained MHC sequences were much more similar to the MHC sequences of *R. bieti* and its closely related species (such as *R. roxellana*) than to those of humans, we considered that the amplified sequences were those of the studied primates rather than humans.

### 2.5. Data Analysis

#### 2.5.1. Genetic Diversity

To examine the genetic diversity of nine microsatellites, we used Cervus V3.0.6 [46] to calculate allelic richness (*A*_R_), observed heterozygosity (*H*_O_), expected heterozygosity (*H*_E_), the effective number of alleles (*A*_E_), polymorphism information content (*PIC*), the and frequency of null alleles (Null). Deviations from Hardy–Weinberg equilibrium (HWE) and the inbreeding coefficient (*F*_IS_) were obtained using Genepop V4.7.0 [47]. For MHC genes, the number of alleles (A), *H*_O_, *H*_E_, and *A*_E_ were calculated using GenAlEx 6.5 [48], *PIC* was calculated using Cervus V3.0.6 [46], HWE and *F*_IS_ were obtained using Genepop V4.7.0 [47] and nucleotide diversity (*Pi*) was calculated using the program DnaSP V6 [49]. Bonferroni correction accounted for potential type I errors resulting from multiple tests.

#### 2.5.2. Selective Pressure Analysis

We calculated the *ω* (the ratio of non-synonymous to synonymous substitutions, *d*_N_/*d*_S_) at antigen-binding sites (ABSs), non-antigen-binding sites (non-ABSs), and all amino acid sites in the exon 2 region using the Nei–Gojobori method with Jukes–Cantor correction [50] in MEGA V7 [51]. One thousand bootstrap replicates were used to obtain standard errors. The ABSs and non-ABSs of *R. bieti* were presumed through the structure of human HLA exon 2 [52]. In the CODEML program of PAML V4.7 [53], *ω* and positive selection sites were obtained with the maximum likelihood method. Based on the different selection intensities among sites, six models (M0: a single *ω* (*d*_N_/*d*_S_) for all codons; M1a: nearly neutral, with two site classes of 0 < *ω*_0_ < 1 and *ω*_0_ = 1 for all branches; M2a: positive selection (a proportion of codons with *ω* > 1); M3: *ω* is a simple discrete distribution; M7: nearly neutral (0 < *ω* ≤ 1), with a variation approximately β-distribution; and M8: close to neutral (0 < *ω* ≤ 1), with a variation approximately β-distribution) were used. They were compared with likelihood ratio tests in PAML V4.7 [53].

#### 2.5.3. Phylogenetic Analysis

We constructed phylogenetic trees of *DQA1*, *DQB1*, *DRB*, and *DPB1* with maximum likelihood (ML) and Bayesian methods. Orthologous sequences and an outgroup sequence at each locus were obtained from the NCBI (https://www.ncbi.nlm.nih.gov, accessed on 12 January 2022) (Table S2). We uniformly used *Mumu*-*H2*-*Aa* (from *Mus musculus*; GenBank accession number: NM_010378.3) as the outgroup.The optimal models for ML and Bayesian trees were determined using jModelTest V2.0 [54] and MrModeltest V2 [55], respectively. Then, according to the best models selected, the ML tree was constructed in PHYML V3.0 [56]. At the same time, the Bayesian tree was established with the Markov Chain Monte Carlo (MCMC) (1000 generations) method [57] in MrBayes 3.2 [58]. The reliability of topology structures was calculated through 1000 bootstrap replications for the ML tree and posterior probability for the Bayesian tree.

## 3. Results

### 3.1. MHC Allele Assignment

We isolated sequences of two *DQA1* (246 bp and 249 bp), two *DQB1* (270 bp), three *DPB* (262 bp), and four *DRB* (*DRB1*: 270 bp; *DRB5*: 270 bp and 267 bp) from 48 individuals of the targeted population with 11 different MHC sequences. Five to ten of them were isolated from each individual, with an average of 7.58. Each sequence was aligned with that of the whole genome of *R. bieti* (GenBank accession number: GCA_001698545.1 [36]. Each sequence of *DQA1*, *DQB1*, and *DPB* matched one fragment of the entire genome sequence of *R. bieti* (GenBank accession number: GCA_001698545.1 [36] (at best, *DQA1*: ranging from 360,642 to 360,635; *DQB1*: 343,806 to 344,099 bp; and DPB: 391,331 to 391,625). We made sure that the primers of *DQA1*, *DQB1*, and *DPB* were primers for single-locus amplification. Four *DRB* sequences matched two fragments of the *R. bieti* genome sequence (ranging from 531,423 to 344,099 and 476,943 to 477,260, respectively), indicating that two *DRB* loci, *DRB1* and *DRB5*, were amplified.

According to the nomenclature [59], these sequences were labelled as Rhbi-DQA1*01-02 (GenBank accession number: PP889557, PP889558), Rhbi-DQB1*01-02 (GenBank accession number: PP889559, PP889560), Rhbi-DPB1*01-03 (GenBank accession number: PP889561, PP889562, PP889563), Rhbi-DRB1*01-02 (GenBank accession number: PP889564, PP889565) and Rhbi-DRB5*01-02 (GenBank accession number: PP889566, PP889567). Each allele could be translated into a unique amino acid sequence. Two alleles lost three nucleotides, resulting in the deletion of one amino acid residue (the 51st residue of DQA1*01 and the 73rd residue of DRB5*01); such a deletion did not cause a variation in the reading frame.

### 3.2. Genetic Variation at Microsatellites and MHC Genes

The genetic diversity parameters of nine polymorphic microsatellite loci from 48 non-repetitive individuals were statistically analyzed (Table 1). The number of alleles ranged from three to nine, averaging 5.444 per microsatellite locus. The average values of *H*_E_ and *H*_O_ were higher than 0.5 (*H*_E_ = 0.565; *H*_O_ = 0.549), indicating a high level of heterozygosity in microsatellites. The mean value of *PIC* was 0.520 (ranging from 0.242 to 0.741), suggesting a high level of polymorphism. Only the locus *D11S2002* deviated significantly from HWE.

As for the five MHC loci, the average values of *H*_E_ and *H*_O_ were 0.500 (from 0.474 to 0.528, Table 2) and 0.513 (from 0.438 to 0.604, Table 2), respectively, indicating a high level of MHC heterozygosity. The average value of *PIC* was 0.383 (between 0.362 and 0.433, Table 2), indicating a moderate polymorphism of the MHC genes. The value of *Pi* is from 0.079 to 0.146, with an average of 0.102 (Table 2). None of the MHC genes deviated from HWE.

### 3.3. Positive Selection

The selection parameter *ω* (*d*_N_/*d*_S_) was calculated for each MHC locus’s ABS, non-ABS, and entire collection of amino acid sites (Table S3). For the ABSs and all of the amino acid sites of *DQB1*, ABSs, non-ABSs and all of the amino acid sites of *DRB1*, and ABSs of *DRB5*, *ω* was greater than one, without reaching a significant statistic level (*DQB1*: ABS: *ω* ≥ 1, *p* = 0.498 and All: *ω* = 1.494, *p* = 0.438; *DRB1*: ABS: *ω* = 1.656, *p* = 0.559, non-ABS: *ω* = 1.262, *p* = 0.747, and All: *ω* = 1.466, *p* = 0.231; *DRB5*: ABS: *ω* = 3.267, *p* = 0.191) (Table S3). Concerning other sites of *R. bieti* MHC genes, *ω* is less than one, which is not statistically significant (Table S3). These results suggest neutral selection rather than positive selection of *R. bieti* MHC genes.

Amino acid residues under significant positive selection were found in *DQB1*, *DRB1*, and *DRB5* loci with PAML V4.7 (Table 3). Variant codon evolution models were selected with the CODEML program based on AIC criteria, indicating that the M2a, M3, and M8 models matched MHC better than others. Under model M2a, two *DRB1* sites (73Y and 81F) were exposed to significant selection. With model M3, 2 *DQB1* sites (21L and 75R), 2 *DRB1* sites (73Y and 81F), and 11 *DRB5* sites (4Q, 8L, 20Q, 23E, 25Y, 32F, 42F, 46S, 52E, 55N, and 69R) were identified, which demonstrated a significant positive selection. With model M8, 21L of *DQB1* and 73Y and 81F of *DRB1* were detected, expressing a significant positive selection. Moreover, most of these sites are ABSs, indicating that functional sites have predominantly undergone positive selection.

### 3.4. Trans-Species Evolution

**Figure 1:**
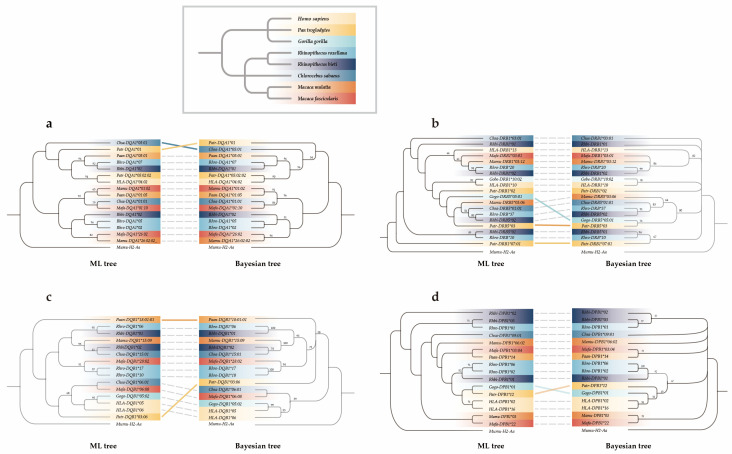
Phylogenetic relationships of the five MHC genes of *R. biet*, conducted using the Bayesian approach and the maximum likelihood method. (**a**) *Rhbi-DQA1*, (**b**) *Rhbi-DRB1* and *Rhbi-DRB5*, (**c**) *Rhbi-DQB1*, and (**d**) *Rhbi-DPB1*. The inset presents a phylogenetic tree illustrating the evolutionary relationships among *R. bieti* and its closely related species (*M. fascicularis*; *M. mulatta*; *R. roxellana*; *Chlorocebus sabaeus*; *Gorilla gorilla*; *Pan troglodytes*; and *Homo sapiens*). The color block of the branch corresponds to the inset and denotes the species from which orthologous sequences come. Values on the branch are the Bayesian tree’s posterior probability and the ML tree’s support rate. The dashed line indicates that the topology of the two trees in this branch has not changed, and the solid line indicates that the topology of the two branches has changed.

Bayesian and ML trees were constructed to investigate the phylogenetic relationships of MHC genes between *R. bieti* and other primates (Figure 1). The reconstructed phylogenetic relationships show that the allelic relationships at all five loci are inconsistent with the species relationship; alleles from different species were intermixed. There was not a clear branch for *R. bieti* alleles in the phylogenetic tree. For example, Rhbi-DQA1*02 was more similar to the alleles from *M. fascicularis*, *M. nemestrina*, and *R. roxellana* than Rhbi-DQA1*01 (Figure 1a). Similarly, Rhbi-DQB1*01 was clustered together with Rhro-DQB1*06 rather than Rhbi-DQB1*02 (Figure 1c). The results demonstrated that the phylogeny of *R. bieti* MHC sequences is consistent with trans-species evolution.

## 4. Discussion

**Figure 2:**
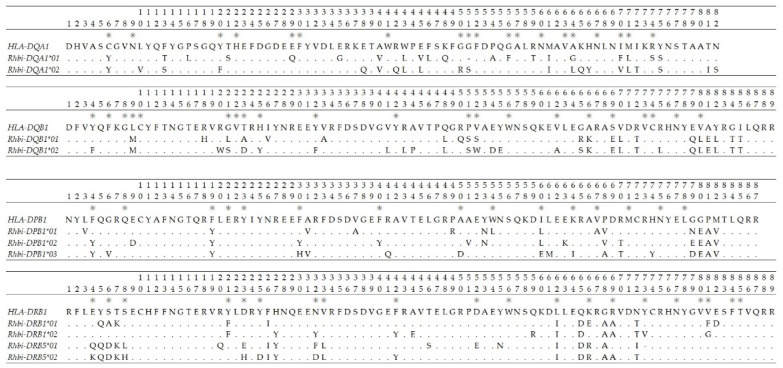
The amino acid sequence of the second exons of five MHC genes in *R. bieti*. “.” is identical amino acids as the consensus sequence and “*” is putative antigen-binding sites inferred from the HLA equivalents [48].

We measured genetic variation at both neutral (nine microsatellites) and adaptive loci (five MHC genes) in a wild *R. bieti* population (XGQ). A total of 11 different MHC sequences were amplified from 48 individuals. Microsatellites displayed high levels of genetic polymorphism and heterozygosity (*PIC* = 0.520; *H*_O_ = 0.549; and *H*_E_ = 0.565) (Table 1) but this was moderate for the former and high for the latter at MHC loci (*PIC* = 0.383; *H*_O_ = 0.513; and *H*_E_ = 0.500) (Table 2). Furthermore, nucleotide diversity was high for each MHC locus (*Pi*_average_ = 0.102). We also identified several amino acid sites under significant positive selection in *DRB1*, *DRB5*, and *DQB1* (Table 3), despite no evidence of substantial positive selection being found at the ABSs, non-ABS, and whole region of exon 2 of five MHC loci. Trans-species evolution was observed in the MHC sequences of *R. bieti* and its close-related species (Figure 2).

### 4.1. Genetic Diversity

The MHC region is one of the most variable regions in the vertebrate genome [60]; therefore, the genetic diversity of MHC is typically more significant than that of the entire genome. Instead, our data indicate that the observed and the expected heterozygosity of MHC in *R. bieti* were lower than those of microsatellites (microsatellites: *H*_O_ = 0.549 and *H*_E_ = 0.565; MHC: *H*_O_ = 0.513 and *H*_E_ = 0.500). However, MHC’s heterozygosity is higher than that of the whole genome (*H*_E_ = 0.034%) [33,61,62]. Such a phenomenon might be caused by an overestimation stemming from the application of microsatellites rather than genome-wide heterozygosity; the loci of microsatellites were studied, but those exhibiting low levels of polymorphism were eliminated from the analysis. One microsatellite locus has a strongly positive *F*_IS_, which may be due to the existence of null alleles that cause the inbreeding coefficients to be overestimated (*D1s207*: *F*_IS_ = 0.215, Null = 0.134)

The MHC diversity of *R. bieti* is much lower than that of the golden snub-nosed monkey (*R. roxellana*) (*H*_O_ = 0.63; *H*_E_ = 0.62; and *PIC* = 0.57), and so is the number of alleles (*R. roxellana*: 42 *DRB* alleles, 6 *DPB1* alleles, 9 *DQA1* alleles, and 17 *DQB1* alleles) [17,22,41,63,64,65]. The reasons for this phenomenon are rather complicated. First, in terms of demographic history, *R. roxellana* has undergone two bottleneck periods (approximately 2 mya and 0.10–0.40 mya) and two population expansions (approximately 1.00 mya and 0.05–0.07 mya) [36]. In contrast, the population size of *R. bieti* has continuously decreased [36]. Thus, *R. roxellana* has experienced a higher accumulation of genetic variation due to the two population expansions [36]. Second, in terms of distribution range and population size, *R. roxellana* is distributed across three distinct areas, the Minshan and Qionglai Mountains, the Qinling Mountains, and the Shennongjia National Nature Reserve [26], with a population size of approximately 22,500 individuals [66]. However, *R. bieti*, around 3000 individuals, is confined to a narrow region between the Lancang and Jinsha Rivers in the middle of the Yunling Mountains [28]. Thus, genetic drift may be intensified due to the small population and geographic isolation, causing the reduction in MHC’s genetic variation [67]. Finally, regarding pathogen pressure, as mentioned above, *R. roxellana* lives in heterogeneous habitats, making it face diverse parasite pressures [68], driving the divergence of MHC sequences. In contrast, *R. bieti* lives in relatively homogeneous habitats and may face homogenized parasite environments, resulting in less diverse MHC sequences. Furthermore, *R. bieti* inhabits the mountains between 3800 and 4300 m above sea level, where snow persists throughout the year, different from the mountains inhabited by *R. roxellana*, from 1400 to 3300 m [26]. Previous studies have indicated an increasing trend of pathogen richness and diversity from colder to warmer areas following the increasing genetic diversity of MHC [69,70,71,72]. The higher altitude and the lower temperature (the *R. bieti* habitat has an average temperature of 7.5 °C [73], while that of *R. roxellana* in the Qinling Mountains is 10.2 °C [74]) have caused *R. bieti* to face reduced pathogen pressure. We only identified 16 amino acid sites that are under significant positive selection in the exon 2 sequences of *Rhbi-DRB1*, *DRB5*, and *DQB1*, and no significant positive selection was found at the ABSs, non-ABS, and whole region of exon 2. In contrast, *R. roxellana* has 68 amino acid sites under significant positive selection [22,43,64], possibly due to more intense selection pressures. Another study reveals that the MHC variation of the Galápagos hawk (*Buteo galapagoensis*), an island species, is lower compared to that of the Swainson’s hawk (*B. swainsoni*), a mainland species; a relaxed selection pressure is produced by the lower parasite diversity on the islands compared to the mainland [75]. The same phenomenon was also found in the sympatric Lake Malawi cichlids—goldbreast zebra cichlid (*Pseudotropheus fainzilberi*) and red zebra cichlid (*P. emmiltos*)—where a substantial amount of variance (26%) in infecting parasite communities is explained by variation among collection sites. In other words, different environments have shaped different parasite pressures [76]. Even among the populations within the same species, heterogeneous pathogen pressure can also lead to MHC variation, which has been reported in the great snipe (*Gallinago media*), guppy (*Poecilia reticulata*), Omei tree frog (*Rhacophorus omeimonis*), house sparrow (*Passer domesticus*), and tuatara (*Sphenodon* spp.) [19,77,78,79,80]. Overall, genetic drift and relaxed selection pressure have shaped the MHC diversity of *R. bieti.*

Contrary to theoretical expectations, we observed high heterozygosity at both microsatellite and MHC loci in a small isolated population. The following reasons cause this phenomenon: (1) although the populations are isolated, male disperse between the populations occurs [30], promoting gene flow among populations to maintain a high level of heterozygosity [17]; (2) MHC genes are subject to pathogen-mediated balancing selection [81], and thus have high levels of genetic variation to cope with diverse pathogens; and (3) the microsatellites used are polymorphic ones, screened among loci, and those with low polymorphism were eliminated prior to analysis.

### 4.2. Historical Balancing Selection

We found two pieces of evidence indicating that balancing selection has acted on the MHC variation of *R. bieti.*

First, the analysis of six random-site models in PAML V4.7 with the maximum likelihood method revealed the existence of positive selection. This finding demonstrated that the models with selection (M2a, M3, and M8) were more suitable for MHC sequences than those without selection (Table 3). Two sites (73Y and 81F) of *DRB1* were exposed to significant selection under all three models, while thirteen additional sites (21L and 75R of *DQB1*, 4Q, 8L, 20Q, 23E, 25Y, 32F, 42F, 46S, 52E, 55N, and 69R of *DRB5*) under model M3 and one site (21L of *DQB1*) under model M8 were under positive selection (Table 3). Among the 15 sites subject to positive selection, 11 were ABSs, and three were adjacent to ABSs (Figure 2), suggesting that selection often occurs in functionally essential domains [22,60,82]. It is possible that single-population sampling in this research may have limited the detection of both novel MHC sequences and positive selection sites.

Second, trans-species polymorphisms, the retention of alleles among species for extended evolutionary history, indicates past balancing selection [83,84,85]. Our results indicated that the sequences of the MHC genes did not cluster according to the phylogenetic relationships of the species. Similarly, trans-species polymorphism patterns of MHC gene have been detected in numerous vertebrate taxa, such as Dabry’s sturgeon (*Acipenser dabryanus*) [86], Japanese ranidae frog (*Rana japonica*) [87], loggerhead sea turtle (*Caretta caretta*) [88], common buzzard (*Buteo buteo*) [89], golden jackal (*Canis aureus*) [90], and Cheirogaleidae [91]. This research presents clear phylogenetic evidence of the trans-species evolution of MHC sequences across *R. bieti*, *R. roxellana*, and *Chlorocebus sabaeus* (Figure 1). That indicates that, due to balancing selection, some allelic lineages have been preserved, and specific alleles shared among species are more ancient than the diversification time of species or even families.

In order to seek additional evidence for balancing selection on MHC genes, the scope of research should be broadened. First, the genetic differentiation patterns expected from MHC and microsatellites should be different. On the one hand, if the pathogen pressure among populations is similar, the genetic differentiation level of MHC genes should be smaller compared to microsatellites. On the other hand, spatially and temporally fluctuating selection may have shaped a more robust population genetic structure of MHC genes compared to microsatellites [81,92]. In addition, adverse frequency-dependent selection can enhance the effective migration rate of rare alleles among populations and reduce the genetic differentiation level of MHC [92,93]. Rare alleles carried by the immigrants can be found in their offspring in a heterozygous genotype through breeding with locals, which will confer a significant fitness advantage and increase the frequency of the rare alleles [92,93,94]. Second, the association between MHC variation and fitness (such as parasitic resistance, juvenile survival, adult lifespan, adult breeding success, etc.) can be detected. For example, under the heterozygote advantage hypothesis, a positive correlation between MHC heterozygosity and fitness can be observed; under the rare-allele advantage hypothesis, when MHC alleles are not in equilibrium, selection will favor rare alleles over specific common alleles [95].

## 5. Conclusions

We investigated the genetic variation of the black-and-white snub-nosed monkey (*R. bieti*) by integrating adaptive MHC genes and neutral microsatellites. The results indicated that neutral loci of *R. bieti* exhibit high heterozygosity and polymorphism, while MHC genes display high heterozygosity and moderate levels of polymorphism. Evidence suggested that historical balancing selection might have maintained the MHC polymorphism in *R. bieti*. Further studies incorporating MHC genes with fitness indicators and expanding the geographic range could enhance our understanding of the conservation genetics of this species and the effects of balancing and neutral selection on small and isolated populations. In addition, the gene flow caused by male dispersal among populations may result in a high degree of heterozygosity in this small isolated population, so it is essential to establish ecological corridors and improve habitat connectivity to facilitate effective dispersal between the populations.

## Figures and Tables

**Table 1:** Summary of microsatellite variation.

Locus	*A*_R_	*A*_E_	*H*_O_	*H*_E_	*PIC*	*F*_IS_	Null	HWE
*GM108*	5	3.386	0.771	0.712	0.652	−0.084	−0.045	NS
*D17S1290*	4	1.554	0.354	0.360	0.335	0.017	−0.002	NS
*GM109*	9	2.794	0.667	0.649	0.608	−0.028	−0.010	NS
*D11S2002*	7	4.455	0.652	0.784	0.741	0.170	0.079	***
*D1S533*	6	3.949	0.792	0.755	0.708	−0.05	−0.038	NS
*D6S474*	3	1.349	0.250	0.262	0.242	0.045	0.003	NS
*D1s207*	5	2.100	0.417	0.529	0.465	0.215	0.134	NS
*GM214*	7	2.636	0.604	0.627	0.567	0.037	0.026	NS
*D6S493*	3	1.647	0.438	0.407	0.363	−0.075	−0.045	NS
Average	5.444	2.705	0.549	0.565	0.520	−0.047	0.011	

*A*_R_: allelic richness; *A*_E_: effective number of alleles; *H*_O_: observed heterozygosity; *H*_E_: expected heterozygosity; *PIC*: the polymorphism information content; *F*_IS_: inbreeding coefficients; Null: frequency of null alleles; HWE: Hardy–Weinberg equilibrium; ***: significant; NS: not significant.

**Table 2:** Genetic diversity at MHC loci in *Rhinopithecus bieti*.

Locus	*A*	*Pi*	*A*_E_	*H*_O_	*H*_E_	*PIC*	*F*_IS_	HWE
*DQA1*	2	0.146	1.900	0.521	0.474	0.362	−0.070	NS
*DQB1*	2	0.107	1.999	0.604	0.500	0.375	−0.227	NS
*DRB1*	2	0.074	1.999	0.438	0.500	0.375	−0.054	NS
*DRB5*	2	0.079	1.999	0.438	0.500	0.375	0.117	NS
*DPB1*	3	0.106	2.121	0.563	0.528	0.426	−0.023	NS
Average	2.200	0.102	2.003	0.513	0.500	0.383	−0.051	

*A*: number of alleles; *Pi*: nucleotide diversity; *A*_E_: effective number of alleles; *H*_O_: observed heterozygosity; *H*_E_: expected heterozygosity; *PIC*: the polymorphism information content; *F*_IS_: inbreeding coefficients; HWE: Hardy–Weinberg equilibrium; NS: not significant.

**Table 3:** The codon evolution model and positive selection sites of each MHC locus were analyzed by the maximum likelihood method.

Locus	Model	#*p*	Log Likelihood	Estimate Parameters	Positively Selected Sites
*DQA1*	M0 (one ratio)	1	−458.470	*ω*_0_ = 0.749	None
	M1a (nearly neutral)	2	−458.194	*p*_0_ = 0.250 (*p*_1_ = 0.750)	Not allowed
	M2a (positive selection)	4	−457.457	*p*_0_ = 0.965, *p*_1_ = 0.000 (*p*_2_ = 0.035) *ω*_2_ = 17.868	54F, 62G
	M3 (discrete)	5	−457.457	*p*_0_ = 0.000, *p*_1_ = 0.965 (*p*_2_ = 0.035) *ω*_1_ = 0.678, *ω*_2_ = 17.868	54F, 62G
	M7 (beta)	2	−458.209	*p* = 0.039, *q* = 0.014	Not allowed
	M8 (beta and omega)	4	−457.457	*p*_0_ = 0.965 (*p*_1_ = 0.035) *p* = 99.000, *q* = 46.920, *ω*_s_ = 17.878	54F, 62G
*DPB1*	M0 (one ratio)	1	−525.044	*ω*_0_ = 0.328	None
	M1a (nearly neutral)	2	−523.208	*p*_0_ = 0.548 (*p*_1_ = 0.452)	Not allowed
	M2a (positive selection)	4	−523.208	*p*_0_ = 0.548, *p*_1_ = 0.377 (*p*_2_ = 0.075) *ω*_2_ = 1.000	60L
	M3 (discrete)	5	−523.197	*p*_0_ = 0.535, *p*_1_ = 0.226 (*p*_2_ = 0.239) *ω*_1_ = 0.922, *ω*_2_ = 0.922	Not allowed
	M7 (beta)	2	−523.218	*p* = 0.024, *q* = 0.030	Not allowed
	M8 (beta and omega)	4	−523.218	*p*_0_ = 0.999 (*p*_1_ = 0.000) *p* = 0.024, *q* = 0.031, *ω*_s_ = 2.799	60L
*DQB1*	M0 (one ratio)	1	−459.831	*ω*_0_ = 0.731	None
	M1a (nearly neutral)	2	−457.833	*p*_0_ = 0.494 (*p*_1_ = 0.506)	Not allowed
	M2a (positive selection)	4	−454.253	*p*_0_ = 0.947, *p*_1_ = 0.000 (*p*_2_ = 0.053) *ω*_2_ = 52.061	21L, 52S, 55Y, 75R
	M3 (discrete)	5	−454.253	*p*_0_ = 0.000, *p*_1_ = 0.947 (*p*_2_ = 0.053) *ω*_1_ = 0.530, *ω*_2_ = 52.061	**21L**, 52S, 55Y, *75R*
	M7 (beta)	2	−457.834	*p* = 0.005, *q* = 0.005	Not allowed
	M8 (beta and omega)	4	−454.254	*p*_0_ = 0.947 (*p*_1_ = 0.053) *p* = 99.000, *q* = 87.548, *ω*_s_ = 52.068	*21L*, 52S, 55Y, 75R
*DRB1*	M0 (one ratio)	1	−427.018	*ω*_0_ = 0.734	None
	M1a (nearly neutral)	2	−424.528	*p*_0_ = 0.615 (*p*_1_ = 0.385)	Not allowed
	M2a (positive selection)	4	−419.12	*p*_0_ = 0.000, *p*_1_ = 0.931 (*p*_2_ = 0.069) *ω*_2_ = 161.196	5Q, 6A, 7K, 26I, 27H, 32N, 42F, 44A, 59Q, 66E, *73Y*, *81F*, 82D
	M3 (discrete)	5	−418.946	*p*_0_ = 0.050 *p*_1_ = 0.879 (*p*_2_ = 0.071) *ω*_1_ = 0.581, *ω*_2_ = 96.582	5Q, 6A, 7K, **73Y**, **81F**
	M7 (beta)	2	−424.533	*p* = 0.005, *q* = 0.008	Not allowed
	M8 (beta and omega)	4	−418.946	*p*_0_ = 0.929 (*p*_1_ = 0.071) *p* = 99.000, *q* = 71.380, *ω*_s_ = 96.604	5Q, 6A, 7K, 26I, 27H, 32N, 42F, 44A, 59Q, 66E, *73Y*, *81F*, 82D
*DRB5*	M0 (one ratio)	1	−411.081	*ω*_0_ = 0.349	None
	M1a (nearly neutral)	2	−408.087	*p*_0_ = 0.703 (*p*_1_ = 0.297)	Not allowed
	M2a (positive selection)	4	−407.351	*p*_0_ = 0.792, *p*_1_ = 0.000 (*p*_2_ = 0.208) *ω*_2_ = 2.596	23E, 32F, 69R
	M3 (discrete)	5	−407.351	*p*_0_ = 0.624, *p*_1_ = 0.168 (*p*_2_ = 0.208) *ω*_1_ = 0.000, *ω*_2_ = 2.596	**4Q, 8L, 20Q, 23E, 25Y, 32F, 42F, 46S, 52E, 55N, 69R**
	M7 (beta)	2	−408.088	*p* = 0.005, *q* = 0.012	Not allowed
	M8 (beta and omega)	4	−407.351	*p*_0_ = 0.792 (*p*_1_ = 0.208) *p* = 0.005, *q* = 80.070, *ω*_s_ = 2.596	23E, 32F, 69R

*#p* is the number of free parameters in the ω distribution. The parameters in parentheses are not free and should not be calculated. The inferred locus by selection is shown in italics when the posterior probability exceeds 95%. The loci inferred by selection with a test probability >99% are shown in bold.

## Data Availability

Obtained MHC sequences have been uploaded to the National Center for Biotechnology Information (NCBI). The results of genotyping are openly available in FigShare at DOI: 10.6084/m9.figshare.26403073.

## Supplementary Materials

The following supporting information can be downloaded at: https://www.mdpi.com/article/10.3390/ani14152276/s1, Table S1 Information of 11 microsatellite loci in *Rhinopithecus bieti* [38,39]. Table S2 Homologous sequences for *Rhinopithecus bieti* MHC genes from closely related species and an outgroup sequence from *Mus musculus* for phylogenetic reconstruction. Table S3 Rate of non-synonymous substitutions (*d*_N_) and synonymous substitutions (*d*_S_) of five *Rhinopithecus bieti* MHC loci. Table S4 likelihood ratio test of codon evolution for the second exons of five *Rhinopithecus bieti* MHC loci.
