# Supplementary material for: Moderate Genetic Diversity of MHC Genes in an Isolated Small Population of Black-and-White Snub-Nosed Monkeys (Rhinopithecus bieti)
Source: Animals (Basel). 2024 Aug 5;14(15):2276. doi: 10.3390/ani14152276 (PMC11310952; doi:10.3390/ani14152276)
Supplement: Supplementary file 1 [file animals-14-02276-s001.zip › animals-3077054-supplementary.pdf]

Table

Table S1 Information of 11 microsatellite loci in *Rhinopithecus bieti*

Locus	Forward primer sequence (5'-3')	Reverse primer sequence (5'-3')	T_A (°C)	Reference
D1s207	CACTTCTCCTTGAATCGCTT	GCAAGTCCTGTTCCAAGTCT	50	(Liu et al. 2008)
D1S533	CATCCCCCCCAAAAAATATA	TTGCTAATCAAATAACAATGGG	50	(Liu *et al.* 2008)
D2S1326	AGACAGTCAAGAATAACTGCCC	CTGTGGCTCAAAAGCTGAAT	55	(Liu *et al.* 2008)
D6S474	ATCCCAACTCTTAAATGGGC	TTCCATGGCAGAAATTGTTT	52	(Liu *et al.* 2008)
D6S493	ATCCCAACTCTTAAATGGGC	TTCCATGGCAGAAATTGTTT	50	(Liu *et al.* 2008)
D8S505	CAAAAGTGAACCCAAACCTA	AGTGCTAAGTCCCAGACCAA	58	(Liu *et al.* 2008)
D11S2002	CATGGCCCTTCTTTTCATAG	AATGAGGTCTTACTTTGTTGCC	55	(Liu *et al.* 2008)
D17S1290	GCCAACAGAGCAAGACTGTC	GGAAACAGTTAAATGGCCAA	55	(Liu *et al.* 2008)
GM108	CAGCGTAAGCCAGTTGCC	GGAAAAGTCTGAAACCCACGA	51	(Hao et al. 2007)
GM109	GGTGGAGGAGGGCCTAAC	CTGATGTCCATAGGCGACCAT	64	(Hao *et al.* 2007)
GM214	GGGCAACAGAGCGAGACTG	TGCAAAGATGTGAACGGAAAT	58	(Hao *et al.* 2007)

Table S2 Homologous sequences for *Rhinopithecus bieti* MHC genes from closely related species and an outgroup sequence from *Mus musculus* for phylogenetic reconstruction

Species	Allele	GenBank number
Mus musculus	*Mumu-H2-Aa*	NM_010378.3
Rhinopithecus roxellana	*Rhro-DQA1*02*	JQ217108.1
	*Rhro-DQA1*05*	JQ217111.1
	*Rhro-DQA1*07*	JQ217113.1
	*Rhro-DQB1*10*	JQ217125.1
	*Rhro-DQB1*17*	KU184585.1
	*Rhro-DQB1*06*	JQ217121.1
	*Rhro-DPB1*02*	PP871676
	*Rhro-DPB1*06*	PP871680
	*Rhro-DPB1*01*	PP871675
	*Rhro-DRB*10*	JQ863331.1
	*Rhro-DRB*20*	JQ863341.1
	*Rhro-DRB*37*	JQ863358.1
Papio anubis	*Paan-DQA1*01:05*	OP375770.1
	*Paan-DQA1*05:01*	LT607037.1
	*Paan-DQB1*18:01:01*	LT546107.1
Pan troglodytes	*Patr-DQA1*01*	DQ924453.1
	*Patr-DQA1*05:02:02*	LT622882.1
	*Patr-DQB1*03:06*	LT908065.1
	*Patr-DPB1*12*	AB183470.1
	*Patr-DRB1*02*	LR216144.1
	*Patr-DRB1*07:01*	DQ655659.1
	*Patr-DRB5*03*	LR216147.1
Macaca mulatta	*Mamu-DQA1*26:02:02*	OX377330.1
	*Mamu-DQB1*15:09*	HQ215939.1
	*Mamu-DPB1*03*	EF426707.1
	*Mamu-DRB1*03:12*	AM910416.1
	*Mamu-DRB5*03:06*	HM594300.1
	*Mamu-DQA1*01:02*	KC428065
Macaca fascicularis	*Mafa-DQA1*26:02*	AM086060.1
	*Mafa-DQA1*01:10*	AB764109
	*Mafa-DQB1*06:08*	AB764119
	*Mafa-DQB1*28:02*	HG994112.1
	*Mafa-DPB1*03:04*	AB764135
	*Mafa-DPB1*22*	AM086165.1
	*Mafa-DRB1*03:01*	AY340675
Homo sapiens	*HLA-DQA1*04:02*	HF674388.2
	*HLA-DQB1*06*	LR777803.1

	*HLA-DQB1*05*	LR778295.1
	*HLA-DPB1*02*	LT630703.1
	*HLA-DPB1*16*	LT882730.1
	*HLA-DRB1*10*	AJ920405.1
	*HLA-DRB1*13*	AJ920408.1
Gorilla gorilla	*Gogo-DQB1*05:02*	KP872253
	*Gogo-DPB1*01*	KP872242
	*Gogo-DRB5*05:01*	KP872282
Chlorocebus sabaeus	*Chsa-DQA1*01:01*	KA650601
	*Chsa-DQA1*05:01*	KA650600
	*Chsa-DQB1*06:01*	KA650615
	*Chsa-DQB1*15:01*	KA650616
	*Chsa-DPB1*09:01*	KA650617
	*Chsa-DRB1*03:01*	KA650612
	*Chsa-DRB5*01:01*	KA650607

Table S3 Rate of non-synonymous substitutions (d_N) and synonymous substitutions (d_S) of five *Rhinopithecus bieti* MHC loci

Locus		N	d_N	d_S	ω (d_N/d_S)	Z	*P*
	ABS	15	0.244 ±0.090	0.266 ±0.198	0.917	-0.095	0.924
DPB1	Non-ABS	72	0.070 ±0.017	0.157 ±0.050	0.446	-1.628	0.106
	All	87	0.098 ±0.020	0.172 ±0.047	0.570	1.390	0.084
	ABS	19	0.227±0.107	0.228±0.216	0.996	-0.004	0.997
DQA1	Non-ABS	63	0.130±0.030	0.226±0.085	0.575	-0.986	0.326
	All	82	0.150 ±0.031	0.227±0.078	0.661	0.855	0.197
	ABS	21	0.229±0.102	0.000±0.000	≥1	0.004	0.498
DQB1	Non-ABS	68	0.097±0.030	0.108±0.053	0.898	-0.181	0.857
	All	89	0.127 ±0.033	0.085±0.040	1.494	0.778	0.438
	ABS	20	0.207±0.096	0.125±0.118	1.656	0.587	0.559
DRB1	Non-ABS	69	0.053±0.020	0.042±0.032	1.262	0.324	0.747
	All	89	0.085±0.025	0.058±0.032	1.466	0.739	0.231
	ABS	20	0.294±0.112	0.090±0.092	3.267	1.314	0.191
DRB5	Non-ABS	69	0.019±0.011	0.114±0.059	0.167	-1.430	0.155
	All	89	0.073±0.023	0.109±0.047	0.670	0.641	0.262

Table S4 likelihood ratio test of codon evolution for the second exons of five *Rhinopithecus bieti* MHC loci

	Models compared	*df*	Test statistic	Significance(*P*)
DQA1	M2a vs. M1a	2	1.474	> 0.05
	M3 vs. M0	4	2.026	> 0.05
	M8 vs. M7	2	1.504	> 0.05
DPB1	M2a vs. M1a	2	0.000	> 0.05
	M3 vs. M0	4	3.694	> 0.05
	M8 vs. M7	2	0.000	> 0.05
DQB1	M2a vs. M1a	2	7.160	< 0.05
	M3 vs. M0	4	11.156	< 0.05
	M8 vs. M7	2	7.160	> 0.05
DRB1	M2a vs. M1a	2	10.816	< 0.01
	M3 vs. M0	4	16.144	< 0.01
	M8 vs. M7	2	11.174	< 0.01
DRB5	M2a vs. M1a	2	1.472	> 0.05
	M3 vs. M0	4	7.46	> 0.05
	M8 vs. M7	2	1.474	> 0.05

1. Hao Y.L., Liu Z.J., Wu H., Ren B.P., Wei F.W. & Li M. (2007) Isolation and characterization of 11 microsatellite loci for the Sichuan snub-nosed monkey, Rhinopithecus roxellana. Conservation Genetics 8, 1021-4.
2. Liu Z.J., Ren B.P., Hao Y.L., Zhang H.R., Wei F.W. & Li M. (2008) Identification of 13 human microsatellite markers via cross-species amplification of fecal samples from <i>Rhinopithecus bieti</i>. International Journal of Primatology 29, 265-72.
